# Compare three deep learning-based artificial intelligence models for classification of calcified lumbar disc herniation: a multicenter diagnostic study

**DOI:** 10.3389/fsurg.2024.1458569

**Published:** 2024-11-06

**Authors:** Zhiming Liu, Hao Zhang, Min Zhang, Changpeng Qu, Lei Li, Yihao Sun, Xuexiao Ma

**Affiliations:** ^1^ Department of Spine Surgery, The Affiliated Hospital of Qingdao University, Qingdao, Shandong, China; ^2^ Department of Neonatology, The Second Affiliated Hospital and Yuying Children’s Hospital of Wenzhou Medical University, Wenzhou, Zhejiang, China

**Keywords:** calcifed lumbar disc herniation, deep learning, artificial intelligence, MRI, ResNet34

## Abstract

**Objective:**

To develop and validate an artificial intelligence diagnostic model for identifying calcified lumbar disc herniation based on lateral lumbar magnetic resonance imaging(MRI).

**Methods:**

During the period from January 2019 to March 2024, patients meeting the inclusion criteria were collected. All patients had undergone both lumbar spine MRI and computed tomography(CT) examinations, with regions of interest (ROI) clearly marked on the lumbar sagittal MRI images. The participants were then divided into separate sets for training, testing, and external validation. Ultimately, we developed a deep learning model using the ResNet-34 algorithm model and evaluated its diagnostic efficacy.

**Results:**

A total of 1,224 eligible patients were included in this study, consisting of 610 males and 614 females, with an average age of 53.34 ± 10.61 years. Notably, the test datasets displayed an impressive classification accuracy rate of 91.67%, whereas the external validation datasets achieved a classification accuracy rate of 88.76%. Among the test datasets, the ResNet34 model outperformed other models, yielding the highest area under the curve (AUC) of 0.96 (95% CI: 0.93, 0.99). Additionally, the ResNet34 model also exhibited superior performance in the external validation datasets, exhibiting an AUC of 0.88 (95% CI: 0.80, 0.93).

**Conclusion:**

In this study, we established a deep learning model with excellent performance in identifying calcified intervertebral discs, thereby offering a valuable and efficient diagnostic tool for clinical surgeons.

## 1 Introduction

Calcified lumbar disc herniation (CLDH) is a specific subtype of lumbar disc herniation. This condition exhibits a relatively low prevalence rate and its underlying causes remain uncertain (1, 2). Long-term lumbar disc herniation exceeding six months can result in the calcification of the protruded nucleus pulposus (3). Consequently, the calcified nucleus pulposus tissue forms extensive adhesions with the dura mater and nerve roots, thereby posing a risk of these tissue tearing (4). Consequently, a significant number of CLDH patients present with pronounced neurological manifestations, encompassing symptoms such as lumbar and leg numbness, pain, and lower limb weakness. Despite attempts at conservative management involving pharmacological, physical, and restorative interventions, the observed effectiveness is often minimal, prompting surgical intervention (5).

Conventional management of CLDH often involves open surgical procedures (6). Nevertheless, advancements in spinal endoscopy equipment and ultrasonic osteotomy techniques have introduced the possibility of utilizing spinal endoscopic surgery for CLDH treatment. Precise determination of intervertebral disc calcification plays a vital role in devising effective treatment strategies. Presently, diagnosis primarily relies on CT values [ranging from 120.1 to 383.7 HU (7)] and histopathological examination involving the identification of calcification foci through microscopic analysis. However, the use of CT scanning poses potential risks of radiation-induced harm to individuals, including pregnant women (8–10), teenagers and children (11–13), and patients with thyroid diseases (14–16). Therefore, the presence of calcification in intervertebral discs significantly affects the use of surgical instruments, the management of intraoperative risks, the duration of surgery, the postoperative recovery, the management of postoperative pain, and the occurrence of postoperative complications. Consequently, the development of a precise, rapid, and non-invasive tool for identifying calcified intervertebral discs is of paramount significance.

In recent years, significant progress has been made in image recognition technology, thanks to the revival of large-scale annotated datasets (i.e., ImageNet) (17) and deep convolutional neural networks (CNNs) (18). ImageNet is a large-scale visual database that contains millions of annotated images, and the CNN models trained on this database are the cornerstone for significantly improving medical image classification problems (19).

Deep learning, a branch of machine learning, has made significant breakthroughs in recent years, particularly in image, language, and speech understanding. Unlike traditional machine learning methods, deep learning can automatically learn data features without the need for manual feature extraction. Deep learning models can handle various types of data and continue to improve with increasing data volume (20).

Deep learning is a type of machine learning method that uses neural network structures similar to those found in the human brain to learn complex patterns in data. CNN is a specific type of deep learning that is particularly suitable for processing data with grid-like structures, such as images (2D grids) and videos (3D grids) (21, 22).

Deep learning has demonstrated remarkable progress in the diagnosis of thyroid cancer (23), esophageal cancer, gastric cancer (24), and skin cancer (25), rivaling the expertise of experienced radiologists (26, 27). Deep learning, with its ability to analyze complex data, has made significant strides in genomics, offering solutions for predicting genetic risks (28, 29), identifying pathogenic mutations (30, 31), and utilizing biomarkers for early disease diagnosis and monitoring (32–34).

Among the fundamental models extensively employed in CNNs, the Residual Network (ResNet) has exhibited commendable performance in both object detection and image classification tasks (35).

The primary aim of this research is to employ deep learning techniques to develop an artificial intelligence-based model that utilizes lumbar spine sagittal MRI images for the precise identification and diagnosis of calcified intervertebral discs, thereby providing assistance to medical practitioners. The performance evaluation of the model will involve utilizing internal test datasets and external validation datasets, where lumbar spine CT scans and intervertebral disc pathology results will serve as the benchmark criteria. The ultimate objective of this study is to furnish clinicians with a rapid and accurate auxiliary diagnostic tool, thereby improving the diagnostic accuracy of calcified intervertebral discs and offering substantial support for disease treatment and patient recovery.

## 2 Materials and methods

### 2.1 Datasets

This study is a retrospective analysis conducted with the approval of the Ethics Committee of our hospital and the informed consent of the patients. The study cohort consisted of 1,224 individuals diagnosed with lumbar disc herniation at our hospital (*n* = 613), Qingdao Municipal Hospital (*n* = 376), and the Second Affiliated Hospital of Wenzhou Medical University (*n* = 235) from January 2019 to March 2024. All enrolled patients underwent routine lumbar MRI and CT scans.

The inclusion criteria were as follows: (1) Diagnosed patients with lumbar disc herniation (including calcified and non-calcified). (2) Lumbar intervertebral disc herniation at the L1-S1 segment, with symptoms lasting at least 3 months, ineffective after conservative treatment or recurrent episodes, and other criteria that meet the indications for surgery. (3) The MRI and CT scans for all patients’ lumbar regions are performed within a 4-week interval. Additionally, patients with calcified lumbar disc herniation must also meet the following criteria: (1) The range of CT value of calcified intervertebral discs in patients with lumbar disc herniation was measured to be between 120.1 and 383.7 Hounsfield units (HU) (7). (2) Histopathological analysis of the excised intervertebral disc tissues confirmed the presence of granular or patchy calcifications when observed under a microscope. Patients who meet all the above three criteria at the same time are finally included in the study.

**Figure 1:**
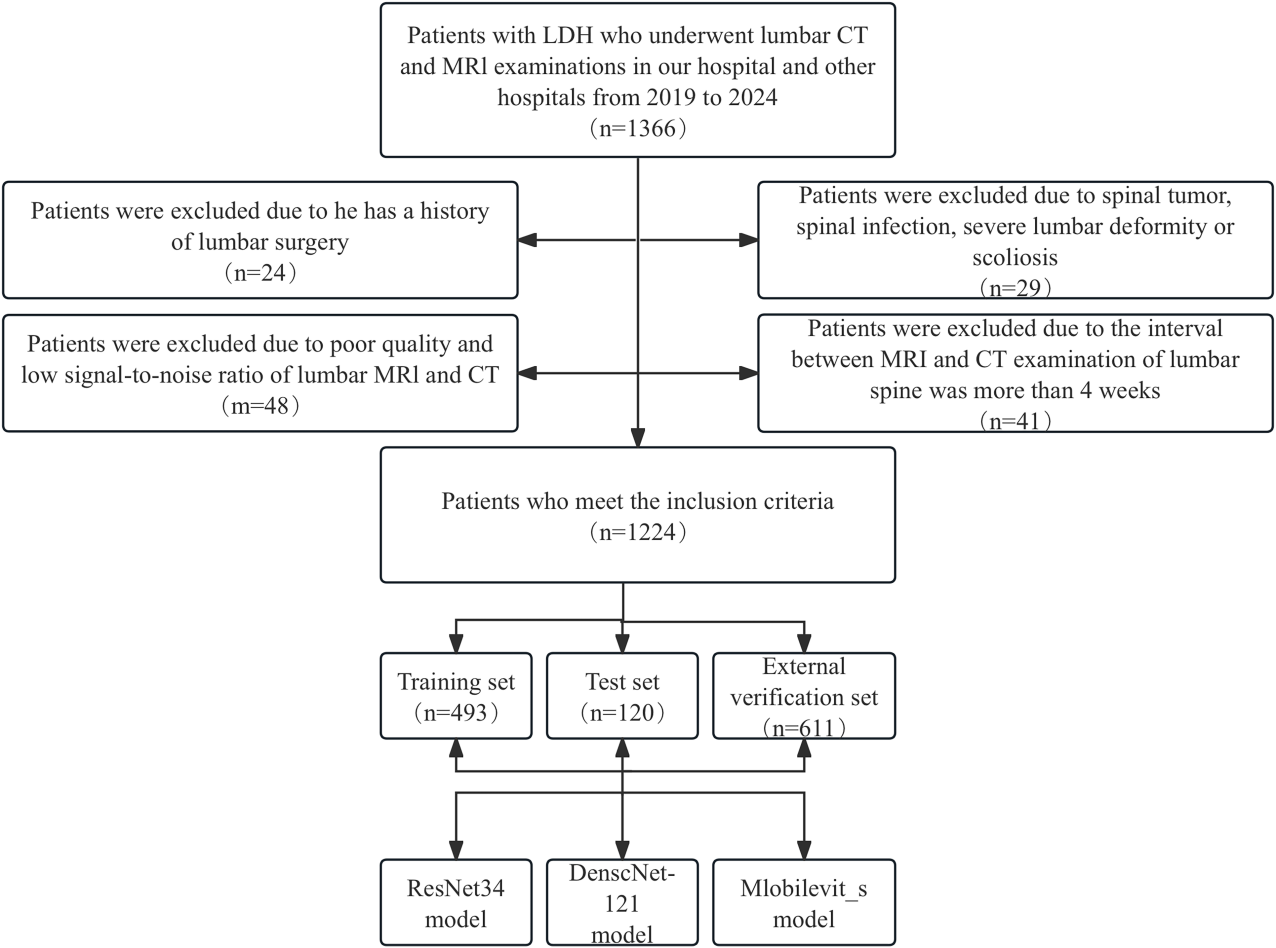
Workflow diagram for developing and evaluating deep learning models for intervertebral disc calcification classification.

The exclusion criteria were as follows: (1) Presence of primary or secondary bone tumors, lumbar spine infections, lumbar spine tuberculosis, and other related conditions. (2) History of previous lumbar spine surgeries. (3) suffering from lumbar scoliosis or severe deformity. (4) Poor image quality or low signal-to-noise ratio. The selection process for patients meeting the inclusion criteria is presented in Figure 1.

In order to verify the accuracy of the deep learning model in clinical practice, 611 patients with age-, sex- and body mass index (BMI)-matched were collected. We employed Propensity Score Matching (PSM) to match 611 patients from the external validation set with 613 patients from the training set, constructing a logistic regression model to predict whether a patient belongs to the training or the external validation set. Using this model, we calculated the probability of each patient belonging to the external validation set, which is the propensity score. The propensity score was then used to match patients in the training dataset with those in the external validation dataset.

### 2.2 Radiological examination

Lumbar spine MRI images were obtained from all patients using a 3.0 T magnetic resonance system, while lumbar spine CT images were acquired using a 128-slice spiral CT scanner.

### 2.3 Image Reading and annotation

**Figure 2:**
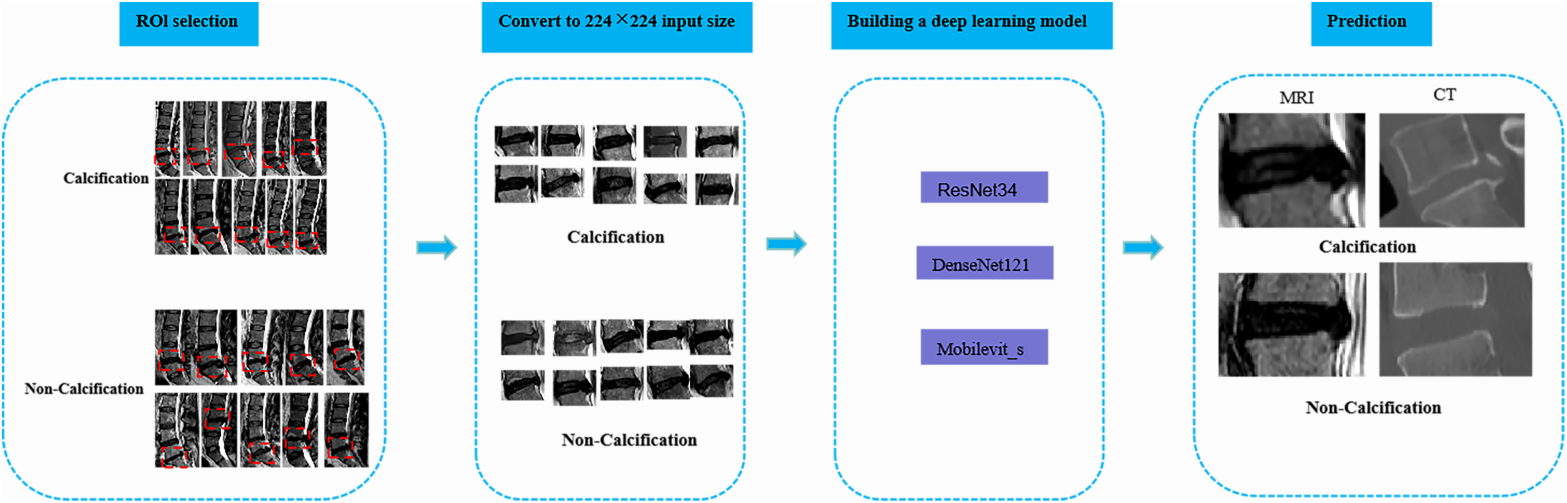
The overall framework of deep learning. To begin with, the protruding intervertebral discs should be accurately delineated on the lateral lumbar MRI images, and the images should be converted to PNG format. Next, the intervertebral disc images need to be resized to 224*224 pixels to prepare them for input into the deep learning model. Subsequently, a classification model for lumbar intervertebral disc calcification will be established based on ResNet-34, DenseNet-121, and Mobilevit_s algorithms. The performance of the model will be evaluated using external validation datasets and the ROC curve. Ultimately, this deep learning model can serve as an assisting tool in the identification of lumbar inervertebral disc calcification.

Two radiology professors with over 10 years of work experience participated in the identification of ROI. They used 3D Slicer (version 5.6.0) to identify the calcified intervertebral disc segments in the mid-sagittal CT images of the patients’ lumbar spine. The presence of intervertebral disc calcification in these segments was determined by a comprehensive assessment that combined the identification results of the radiology professors and the pathological results. Following this, the two radiology professors outlined a rectangular area as the ROI on the mid-sagittal MRI images of the lumbar spine for the corresponding segments of patients with intervertebral disc calcification. This area was centered on the intervertebral disc and extended 0.8–1.2 centimeters above and below. Any discrepancies were resolved through consensus-based discussions. Subsequently, the manually selected images were converted to PNG format with dimensions of 224*224 pixels in order to facilitate subsequent deep learning analysis (Figure 2).

For the non-calcification control group, the specific method for determining the ROI is as follows: In this study, a total of 422 patients were diagnosed with intervertebral disc calcification, with the number and proportion of each segment as follows: L1-2 (*n* = 8, 1.90%), L2-3 (*n* = 7, 1.66%), L3-4 (*n* = 10, 2.37%), L4-5 (*n* = 175, 41.47%), L5-S1 (*n* = 222, 52.60%). Therefore, based on the proportion of each segment in the calcification group, the number and proportion of each segment in the 802 non-calcification patients are as follows: L1-2 (*n* = 15, 1.87%), L2-3 (*n* = 13, 1.62%), L3-4 (*n* = 19, 2.37%), L4-5 (*n* = 333, 41.52%), L5-S1 (*n* = 422, 52.62%). The ROI determination method for the non-calcification group refers to the ROI determination method used for calcification, where on the mid-sagittal MRI image, a rectangular area is drawn with the intervertebral disc at the center, extending 0.8–1.2 cm above and below the disc.

The MR acquisition sequence in this study has a resolution of 0.2 millimeters, an Echo Time (TE) of 20 milliseconds, a Repetition Time (TR) of 1,000 milliseconds, and a Field of View (FOV) of 200 millimeters. The CT acquisition sequence in this study employs a tube voltage of 120 kilovolts (kVp), a milliampere-seconds (mAs) value of 200, a slice thickness of 5 millimeters, a field of view (FOV) of 500 millimeters, and a pitch of 1.0.

### 2.4 Model training

The patients within the development datasets were randomly allocated into training and validation sets at a ratio of 8:2. The training set was utilized to construct the deep learning model for calcified lumbar intervertebral discs, whereas the validation set served to assess the model's performance. The number of patients in each dataset is presented in Table 1.

**Table 1:** Patient characteristics.

Characteristics	Number	Development set	Test set	External validation set	*p* value
Number of patients	1,224	493	120	611	
Sex					*P* = 0.59 (>0.05)
Male	610	253	60	297	
Female	614	240	60	314	
Age	53.34 ± 10.61	53.43 ± 9.88	50.38 ± 10.16	51.61 ± 10.76	*P* = 0.74 (>0.05)
Calcification or not					
Calcification	422	154	40	228	
Non-calcification	802	339	80	383	

For this study, we utilized the ResNet-34 architecture model. The input images were adjusted to a resolution of 224*224 pixels and normalized using Mean = [0.485, 0.456, 0.406] and STD = [0.229, 0.224, 0.225]. We conducted hyperparameter tuning on the optimizer, learning rate, initial weights, image size, and batch size. For the optimizer, we evaluated Stochastic Gradient Descent (SGD) and Adam. For initial weights, we assessed the impact of normal distribution initial weights and selected the best weights from the training epochs (epochs = 150); for the learning rate, the search range for SGD was 0.0001, and default parameters were used for the Adam optimizer; for image size, the search range was 128, 256, and 512 pixels; for batch size, the search range was 2–32. The best performing model was ResNet-34, with the optimal optimizer being Adam (learning rate of 0.0001), using initial weights, and a batch size of 6. Furthermore, to enhance model accuracy, prevent overfitting, and improve training efficiency, we utilized a pre-trained ResNet-34 model on ImageNet as the foundation and fine-tuned it based on this. This strategy can reduce the amount of training data and accelerate the training process. Initializing the weights of the convolutional layers with a normal distribution can help the model escape local optima and improve its generalization capability.

Horizontal flipping is employed to augment and amplify original images, minimizing overfitting. This normalization ensures smooth convergence during training, boosting model stability and efficacy.

### 2.5 Validation of the model

The test datasets served to validate the model's performance. Additionally, external validation datasets from Qingdao Municipal Hospital, and the Second Affiliated Hospital of Wenzhou Medical University (all meeting the same inclusion and exclusion criteria as the training set) was used to assess the robustness of the model. Additionally, the DenseNet-121 and Mobilevit_s models, as well as the human recognition results for images, are compared with the ResNet-34 architecture model to determine the best model. For the manual identification process, the other two radiology experts analyzed the ROI on the previously obtained MRI images to determine whether the segment was calcified, and they were not aware of the CT images or pathological results beforehand.

### 2.6 Statistical analysis

Data analysis and model evaluation were performed using Python (version 3.7.0). The performance of the model was evaluated using ROC curves, AUC, confusion matrices, accuracy, sensitivity, specificity, precision, F1 scores, positive predictive values (PPVs), and negative predictive values (NPVs). The AUC values were calculated using the trapezoidal rule, and the optimal threshold was determined using the maximum Youden index method. The confusion matrices were calculated based on the true positives, false positives, true negatives, and false negatives. Continuous variables were presented as means ± standard deviations and were tested for normality using the Shapiro-Wilk test. Statistical significance was determined using the independent samples *t*-test for continuous variables and the chi-squared test for categorical variables. *P* < 0.05 was considered statistically significant.

## 3 Results

### 3.1 Population characteristics

A total of 1,224 patients were enrolled in this study, comprising 610 males and 614 females, with an average age of 53.34 ± 10.61 years. Out of the 1,224 patients, 802 had non-calcified intervertebral discs, whereas 422 exhibited calcified intervertebral discs (Table 1).

### 3.2 Establishment of deep learning model

After 87 training iterations, no further improvement in the accuracy of calcified intervertebral disc prediction was observed, indicating the completion of the training process.

### 3.3 Deep learning model performance on the different datasets

The ResNet-34 model showed the best performance in the binary classification of distinguishing whether the intervertebral disc is calcified, with an AUC of 0.96 (95% CI: 0.93, 0.99). The DenseNet-121 model achieved an AUC of 0.87 (95% CI: 0.81, 0.93), while the Mobilevit_s model yielded an AUC of 0.82 (95% CI: 0.75, 0.90). The AUC for human recognition is 0.652 (95% confidence interval: 0.587, 0.733).

**Figure 3:**
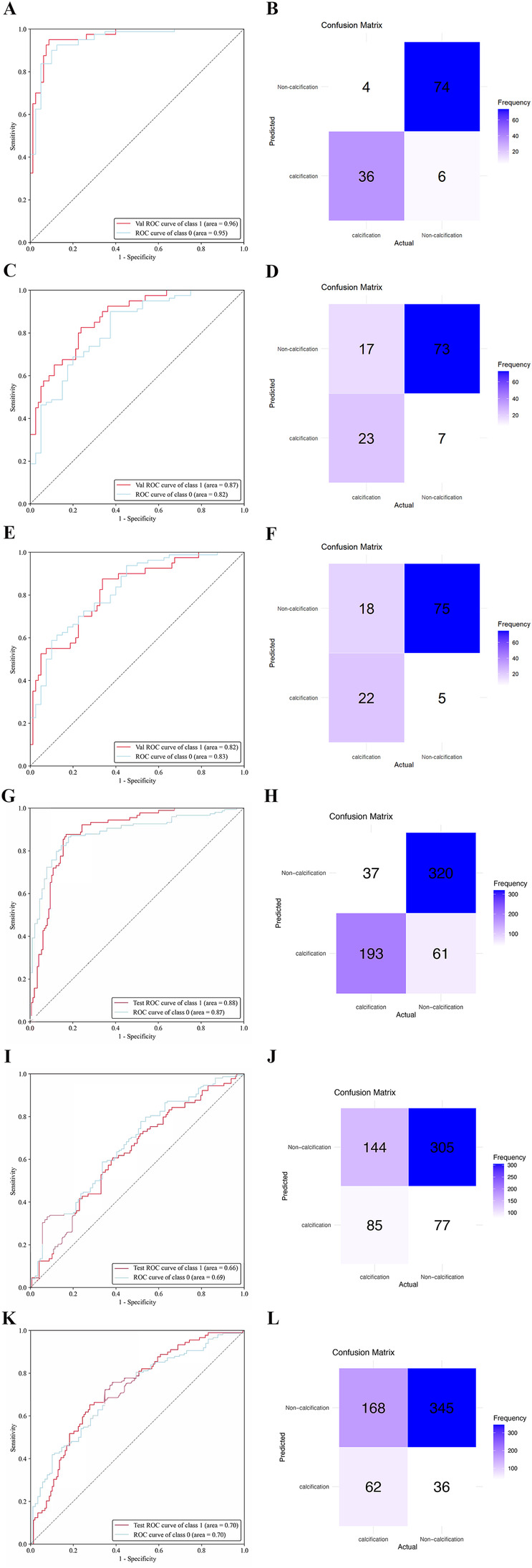
The performance of deep learning models was assessed using internal and external datasets. ROC **(A)** and normalized confusion matrix **(B)** of the ResNet-34 model in the internal test data set. ROC **(C)** and normalized confusion matrix **(D)** of the DenseNet-121 model in the internal test data set. ROC **(E)** and normalized confusion matrix **(F)** of the Mobilevit_S model in the internal test data set. ROC **(G)** and normalized confusion matrix **(H)** of the ResNet-34 model in the external validation data set. ROC **(I)** and normalized confusion matrix **(J)** of the DenseNet-121 model in the external validation data set. ROC **(K)** and normalized confusion matrix **(L)** of the Mobilevit_S model in the external validation data set. Class1 = calcification datasets; Class0 = Non-calcification datasets.

Similar to the testing datasets, the ResNet-34 model also exhibited the highest performance in the external validation datasets, with an AUC of 0.88 (95% CI: 0.80, 0.93). The DenseNet-121 model obtained an AUC of 0.66 (95% CI: 0.57, 0.69), whereas the Mobilevit_s model achieved an AUC of 0.70 (95% CI: 0.66, 0.79) The AUC of human recognition is 0.41 (95% CI: 0.35, 0.58). (Table 2 and Figure 3).

**Table 2:** The performance of deep learning on different datasets and different models.

Data set and model	AUC	Accuracy (%)	Sensitivity (%)	Specificity (%)	Precision (%)	F1 score (%)	PPV (%)	NPV (%)
Testing set								
ResNet34	0.96	91.67	90.00	92.50	85.71	87.80	85.71	94.87
DenseNet121	0.87	80.00	57.50	91.25	76.67	67.71	76.67	81.11
Mobilevit_s	0.82	80.83	55.00	93.75	81.48	65.67	81.48	80.65
Human identification	0.65	70.87	60.98	85.86	78.31	56.93	75.31	66.74
External validation set								
ResNet34	0.88	88.76	87.36	86.74	79.76	80.79	79.77	89.88
DenseNet121	0.66	63.23	36.07	74.63	54.68	44.42	54.28	64.62
Mobilevit_s	0.70	66.54	29.34	60.74	65.46	38.82	60.61	65.64
Human identification	0.41	75.83	40.66	68.92	63.53	52.67	62.36	73.27

AUC, area under curve; PPV, positive predictive value; NPV, negative predictive value.

## 4 Discussion

In this research, we have successfully devised a deep learning model capable of effectively discriminating intervertebral disc calcification. Our model exhibits commendable accuracy and specificity when tested on both internal and external datasets. Its implementation renders formidable assistance to surgeons in precisely diagnosing cases of calcified intervertebral discs and devising appropriate surgical strategies.

Recently, many experts and scholars have attempted to utilize the rapidly developing and widely used artificial intelligence (AI) methods to detect Lumbar disc herniation. Hou et al. have developed a deep learning framework to establish a classifier for diagnosing LDH, which is trained using semi-supervised learning approaches (36). Sustersic et al. have proposed a deep learning model based on convolutional neural networks (CNNs) for the automatic detection and classification of LDH through MRI images (37). Tsai et al. have used the YOLOv3 algorithm to achieve automatic detection of LDH by enhancing and feature-processing MRI images (38). Similar to these articles, this paper also adopts the ResNet architecture and CNN model, and the performance evaluation of the model employs the confusion matrix, accuracy, sensitivity, and ROC curve.

It is undeniable that MRI is less sensitive than CT in determining whether a disc is calcified, because MRI has lower contrast for calcified tissue, while CT scanning can directly display the calcified areas. Despite this, MRI has unique advantages in assessing the disc and its surrounding soft tissues: unlike CT, MRI does not use ionizing radiation, posing a smaller long-term health risk to patients; MRI can provide imaging in any plane, including sagittal, coronal, and axial views, which helps in a more comprehensive assessment of spinal structure; MRI provides better soft tissue contrast, aiding in the differentiation of structures such as the disc, vertebrae, ligaments, muscles, and nerves. The use of this model in conjunction with CT allows doctors to fully understand the patient's condition, thus enabling the creation of more precise and personalized surgical plans.

Alomari et al. conducted a clinical trial demonstrating the favorable effectiveness of T2-weighted lumbar spine sagittal MRI images in evaluating lumbar disc herniation (39). Consequently, we employed T2-weighted lumbar spine sagittal MRI images to construct our deep learning model, aiming to attain enhanced contrast and clearer anatomical features. Previous research has established deep learning models for diagnosing lumbar disc herniation, with their selected ROI encompassing the intervertebral disc as well as the adjacent superior and inferior vertebrae (40). Similarly, in this study, we adopted a similar approach, defining the ROI as the intervertebral disc's central portion along with a 0.8–1.2 cm segment of the surrounding superior and inferior vertebrae.

ResNet-34 (Residual Network-34) is a deep learning model that performs particularly well in image recognition and classification, containing 33 convolutional layers and 1 fully connected layer. A key feature of this network architecture is residual learning, which enables the network to learn the difference between input and output through skip connections, allowing it to be effectively trained even with a deep network. When using ResNet-34, it is typically pre-trained and then fine-tuned for specific tasks such as object classification, target detection, or semantic segmentation.The deep learning model established using ResNet-34 also demonstrated good image recognition performance, capable of distinguishing calcified and non-calcified discs effectively (41–43).

DenseNet-121 is a dense connection network with 121 convolutional layers, each of which is connected to all the previous layers. Due to the dense connections between layers, DenseNet can more efficiently utilize computational resources, reduce the number of parameters, and improve training speed. In addition, DenseNet can also enhance the accuracy of the model, especially in image recognition tasks (44).

MobileViT_s is a lightweight visual transformation model for mobile devices, serving as a variant of the MobileViT model. MobileViT is a model that combines the advantages of convolutional neural networks (CNNs) with the architecture of Transformers. It reduces the number of model parameters and computational complexity by employing depth-wise separable convolutions and mobile windowing mechanisms, while still maintaining the performance benefits of Transformers. Furthermore, the model reduces the size and computational demands, enabling efficient operation on mobile devices (45).

Many studies have demonstrated the effectiveness of deep learning algorithms in the diagnosis of various medical diseases, including breast cancer (46–48), brain tumors (49–51), etc. The results of these studies indicate that deep learning algorithms are promising tools for future medical diagnosis. However, deep learning algorithms also face some challenges in medical image analysis. Firstly, medical image data are typically high-dimensional and complex, requiring substantial computational resources and storage space. Secondly, the annotation process of medical image data often necessitates expertise and technology, consuming a significant amount of time and manpower. Therefore, how to optimize algorithms and improve annotation efficiency is an important research direction in the application of deep learning to medical image analysis. To address these issues, researchers have proposed several methods. For example, transfer learning techniques can be employed to initialize new models with already trained models, thus reducing the amount of data that needs to be annotated (52–54). Additionally, the use of multimodal learning techniques can combine different types of medical image data to enhance the diagnostic performance of models (55–57).

The future development in the field of this study can consider integrating other imaging characteristics for multimodal feature fusion. For instance, the inclusion of lumbar MRI coronal images and x-ray data into the model can be explored to investigate the correlations and complementarity between these different examination modalities, aiming to enhance the performance and accuracy of the model. Additionally, the application of weakly supervised learning methods can be pursued, such as utilizing unlabeled data for self-learning and techniques like transfer learning, to reduce the manual annotation workload and enhance the model's generalization capability. Furthermore, the formation and evolution of calcified intervertebral discs are temporal processes, thus it is worth considering incorporating time-series information into the model. By analyzing the trends and dynamic features of image sequences at different time points, a better understanding of the developmental patterns of calcified intervertebral discs can be gained, thereby improving the predictive ability of the model.

This article inevitably has some shortcomings. First, the number of included patients was limited, with only 1,224 cases. Second, the severity of the disease varied among the enrolled patients, regardless of whether there was intervertebral disc calcification or not, and the degree of lumbar disc protrusion also differed. Third, there was only one external validation datasets in this study, highlighting the importance of additional external validation datasets to verify the established model. The model may need further optimization and adjustment for adaptation to various clinical requirements in actual clinical applications.

## 5 Conclusion

We developed a CNN-based artificial intelligence model for high-accuracy MRI analysis of intervertebral discs, trained on a dataset of calcified and non-calcified scans. It offers surgeons a fast, reliable diagnostic tool, aiding early prediction and description of disc calcification for optimized treatment and outcomes.
